# Predicting the Global Potential Suitable Areas of Sweet Osmanthus (*Osmanthus fragrans*) Under Current and Future Climate Scenarios

**DOI:** 10.1002/ece3.70435

**Published:** 2024-11-05

**Authors:** Yuanzheng Yue, Yingyu Huang, Wei Liu, Xiulian Yang, Lianggui Wang

**Affiliations:** ^1^ State Key Laboratory of Tree Genetics and Breeding Nanjing Forestry University Nanjing China; ^2^ Co‐Innovation Center for Sustainable Forestry in Southern China Nanjing Forestry University Nanjing China; ^3^ College of Landscape Architecture Nanjing Forestry University Nanjing China; ^4^ School of Horticulture and Landscape Architecture Jinling Institute of Technology Nanjing China

**Keywords:** environmental factors, future climates, geographic distribution, MaxEnt model, *Osmanthus fragrans*, suitable habitat

## Abstract

*Osmanthus fragrans* is a valuable landscaping tree that is appreciated worldwide. However, the optimal environmental conditions for *O*. *fragrans* cultivation have yet to be studied in detail, which hinders the preservation of wild resources of this plant and its commercial exploitation. The maximum entropy model was applied to assess the significance of environment variables influencing *O*. *fragrans* distribution. Combining data from 629 global distribution points for *O*. *fragrans*, predictions were made on the potential effects of climate change on the geographical distribution of suitable habitats for this species in the present and the future. The results indicated that *O*. *fragrans* preferred a warm and humid growing environment. Under the current climatic conditions, the potential habitats for *O*. *fragrans* were mostly located in the eastern coastal areas of the continents at medium and low latitudes. The main environmental variables that affected its distribution were the precipitation during the warmest quarter, the temperature seasonality, and the mean temperature of the warmest quarter. The analysis indicated that the continuation of current trends in climate change will result in the further reduction of suitable habitats for *O*. *fragrans* growth, and the global centroid will shift to the southeast. These findings provided insight into the impact of climate change on *O*. *fragrans* habitats, as well as provide guidance for the conservation of wild resources of this species and the breeding of more climate change‐resistant varieties for the future.

## Graphical abstract

The MaxEnt model was used to predict the global distribution of *Osmanthus fragrans*. The precipitation of the warmest quarter, the temperature seasonality, and the mean temperature of the warmest quarter were the most critical factors shaping *O. fragrans* distribution. The suitable areas for *O. fragrans* were concentrated in the east coastal areas of the continents in mid and high latitudes and showed a decreasing trend under the CMIP6 situation.

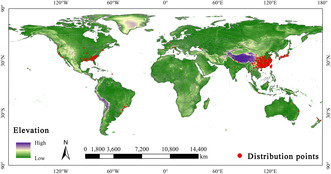

## 1 Introduction

Climate change directly and profoundly affects the distribution patterns of species (Chen et al. 2011). Global climate change has a significant impact on plants, affecting their growth and development, geographic distribution, and population size. The Intergovernmental Panel on Climate Change (IPCC) has reported that global surface temperatures will continue to rise in the future. In general, the increase in global temperatures is accompanied by shifts in species distributions toward more polar regions and/or higher altitudes (Cuena‐Lombraña et al. 2018; Peñuelas et al. 2013). Therefore, identifying potential suitable habitats for a species in future climate scenarios is crucial for the employment of effective strategies to protecting plant biodiversity.

In recent years, the construction of ecological niche models has provided a means to explore the potential geographic distribution patterns and climatic suitability of target species based on their close association with environmental factors and has become a commonly used methodology in many studies addressing climate change (Adhikari et al. 2023; Dunn and Milne 2014). Ecological niche models simulate the actual and potential distribution of species by using the coordinates of their occurrences and the environmental conditions in those locations (Mccormack, Zellmer, and Knowles 2010). With the advancement of climate change research and geographic information technology, various ecological niche models have been established internationally for predicting the distribution of species. Ecological niche models such as MaxEnt, Bioclim, and Climex have become widely used in recent years (Yi et al. 2016). Of these, the MaxEnt, which is based on the maximum entropy algorithm, can provide robust predictions of environmental effects on species distribution with fewer samples (Ahmed et al. 2015; Zhao, Zhang, and Xu 2020). Maximum entropy (MaxEnt) models have been used in the protection of endangered species by providing models of the regulation and preventative management of invasive species (Jiao et al. 2023; Zhang, Shen et al. 2023). The MaxEnt models have also been employed to predict suitable habitats for the cultivation of plants with medicinal or agricultural value (Li et al. 2019; Xu et al. 2020).

**FIGURE 1:**
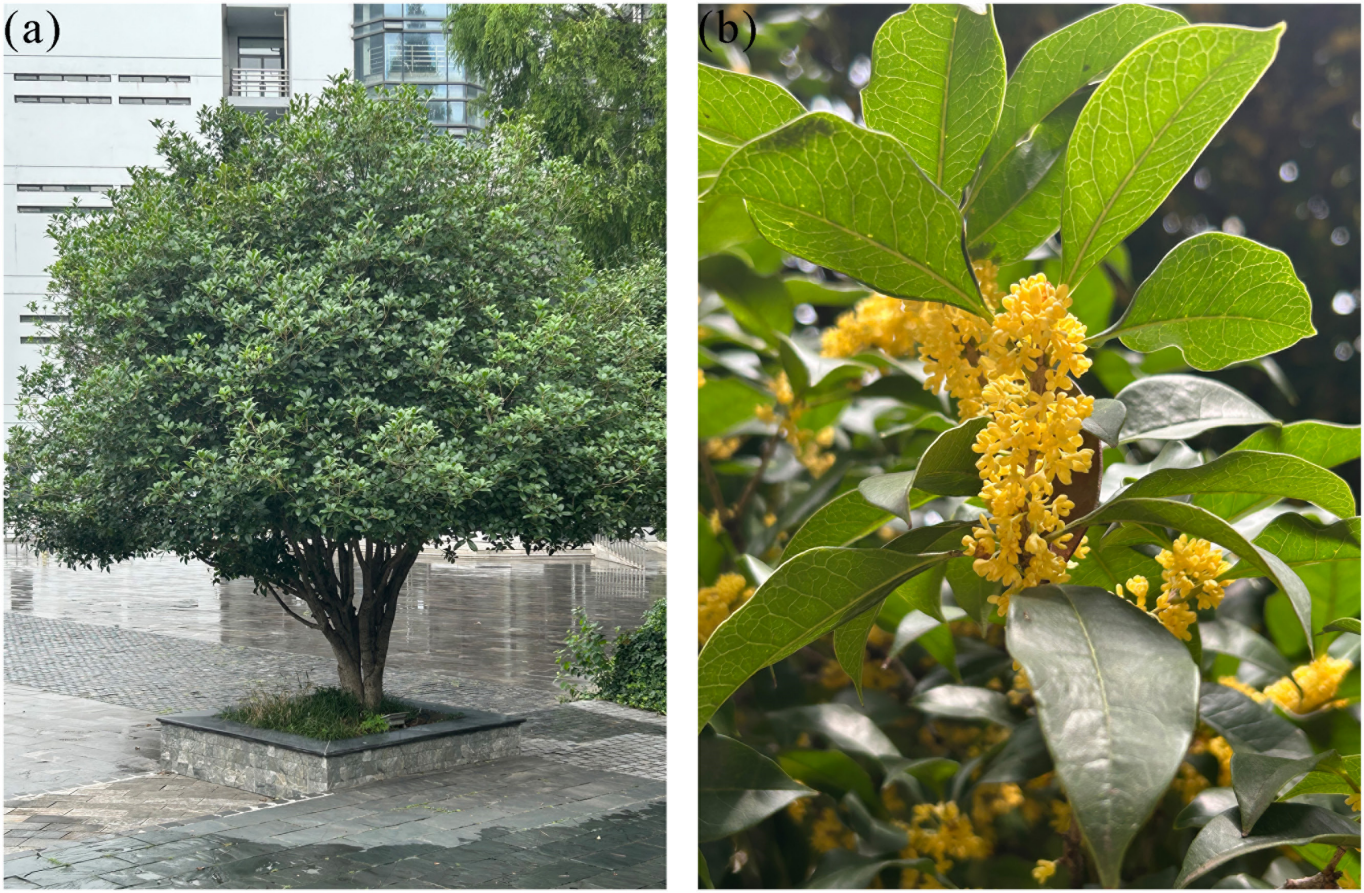
Photograph of *Osmanthus fragrans*. (a) The evergreen *O*. *fragrans* in the garden. (b) The flowers of *O*. *fragrans*.

Sweet osmanthus (*Osmanthus fragrans*) is a small tree species that belongs to the Oleaceae family (Zheng et al. 2023) (Figure 1a). It is indigenous to southwest China and originates from the Chinese‐Himalayan region. China introduced *O*. *fragrans* to Japan for the first time. Around 1771, it was brought from Japan to the United Kingdom and subsequently spread to other European countries and the Americas (Chen and Gong 2022). *O*. *fragrans* has been cultivated in China for many centuries and is considered to have one of the top 10 traditional famous flowers (Zhang et al. 2011). It is appreciated for its tree form and evergreen nature, which makes it an esthetically pleasing choice in gardens, but also for its fragrant flowers (Figure 1b). Additionally, the essential oils extracted from different organs of *O*. *fragrans* have various pharmacological effects (Han et al. 2019; Hung, Tsai, and Li 2012). In China, the natural distribution of *O*. *fragrans* includes subtropical mountainous areas south of the Yangtze River Basin, north of the Nanling Mountains, and east of central Guizhou. The cultivation area of *O*. *fragrans* is restricted in Europe and America due to environmental constraints on growth (Hu et al. 2014). Meanwhile, the overexploitation of wild resources and the weak natural regeneration ability of wild populations have led to a reduction in the distribution of wild *O*. *fragrans*, with a sharp decrease in population size and numbers (Zhang et al. 2011). Therefore, the identification of the main environmental factors affecting *O*. *fragrans* distribution and the prediction of suitable habitats of *O*. *fragrans* in the present and future will help conserve wild genetic resources, but also assist in the application and production of *O*. *fragrans* in the gardening and horticultural industries.

This study used the MaxEnt model to predict current and future potentially suitable habitats for *O*. *fragrans* on a global scale. In this study, we addressed the following question: how will climate change affect the global suitable habitats of *O*. *fragrans*? Therefore, the objectives of this study were as follows: (a) to identify the environmental variables that limit the current and future geographical distribution of *O*. *fragrans*, (b) to predict the potential suitable areas for *O*. *fragrans* under the contemporary climate scenario, and (c) to predict the changes in suitable habitats of *O*. *fragrans* under future climatic scenarios. The study will provide guidelines for the restoration of *O*. *fragrans* populations, the conservation of germplasm resources, and its commercial cultivation.

## 2 Materials and Methods

### 2.1 Species Occurrence Records

**FIGURE 2:**
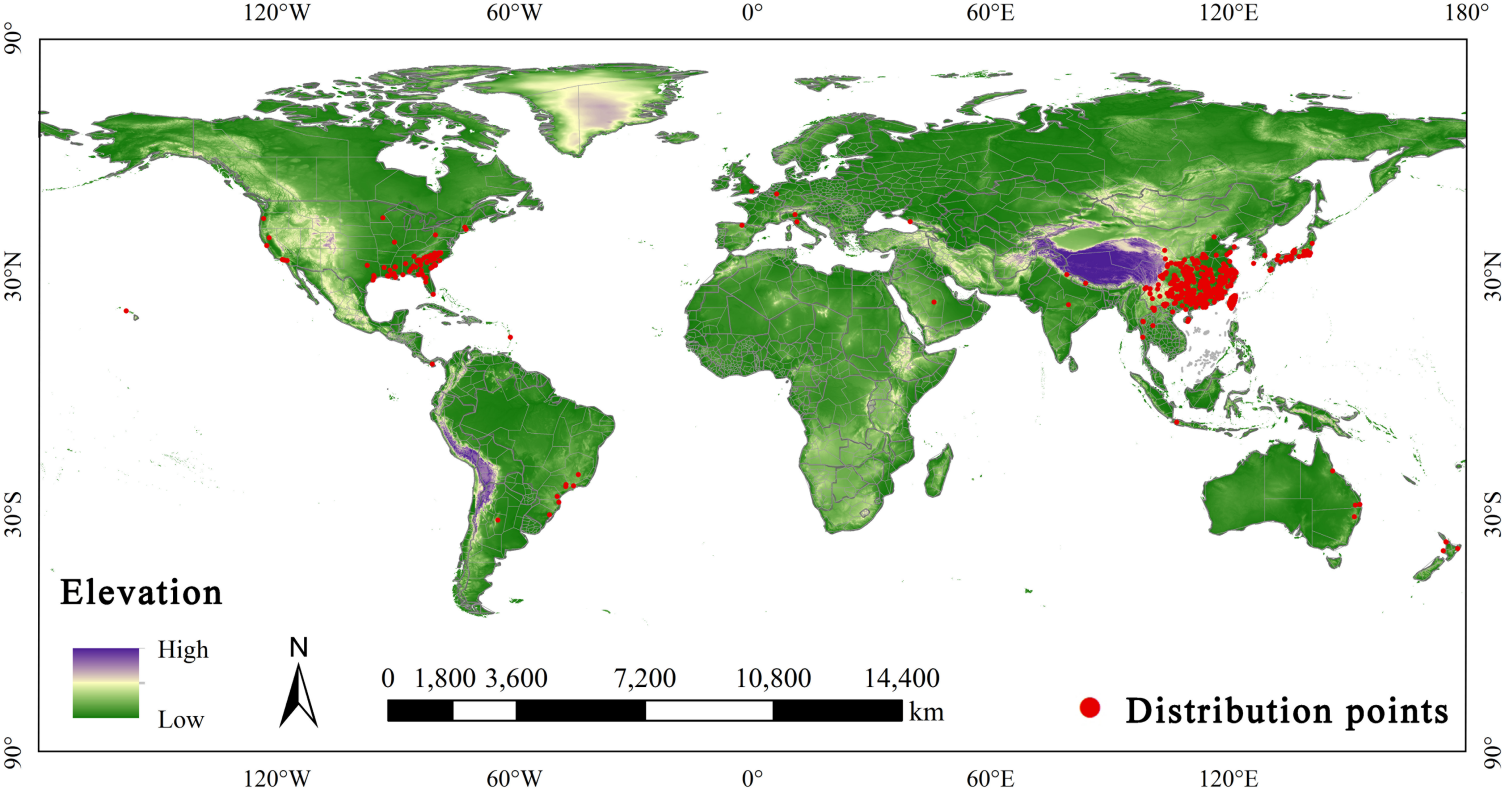
The recorded world‐wide distribution of *O*. *fragrans*.

In this study, occurrence records of *O*. *fragrans* were collected from the Global Biodiversity Information Facility (GBIF, http://www.gbif.org), the China Virtual Herbarium (http://www.cvh.ac.cn), Taiwan Biodiversity Network (http://tbn.org.tw), Atlas of Living Australia (https://www.ala.org.au), and iDigBio (http://idigbio.org). The collected coordinates of *O*. *fragrans* distribution points were placed in a data frame (using Excel), which was filtered by ArcGIS software (Environmental Systems Research Institute, American) for duplicates and coordinates less than 10 km apart in order to avoid sampling bias due to redundancy of coordinates (Aiello‐Lammens et al. 2015; Li, Qi et al. 2022; Parveen et al. 2022). A total of 629 occurrence records were retained to establish the models (Figure 2).

### 2.2 Environmental Parameters

The environmental variables used in this study were downloaded from the WorldClim database (http://www.worldclim.org), which includes 19 bioclimatic variables and three topographic variables (elevation, slope, and aspect), all with a spatial resolution of 2.5 arc‐minutes. Contemporary environmental variables were based on climate data from 1970 to 2000, while the predicted future climate data was selected from the Beijing Climate Center Climate System Model 2 Medium Resolution (BBC‐CSM2‐MR) and the Coupled Model Intercomparison Project (Phase 6; CMIP6) and included the four periods of 2021–2040, 2041–2060, 2061–2080, and 2081–2100 (Wu et al. 2021). There are four predicted scenarios which are based on shared socio‐economic pathways (SSPs) in the future climate data of CMIP6. These scenarios include forcing scenarios ranging from high (SSP5‐8.5), moderate to high (SSP3‐7.0), moderate (SSP2‐4.5), and low (SSP1‐2.6). SSP5‐8.5 is the only scenario with anthropogenic radiative forcing that will reach 8.5 W/m^2^ by 2100; SSP3‐7.0 is a combination of relatively high social vulnerability and anthropogenic radiative forcing that will stabilize at 7.0 W/m^2^ in 2100; SSP2‐4.5 is a scenario that combines moderate social vulnerability and moderate radiative forcing and will stabilize at 4.5 W/m^2^ by 2100; SSP1‐2.6 represents a scenario under the combined effect of low vulnerability, low mitigation pressure, and low radiative forcing, where the radiative forcing will stabilize at 2.6 W/m^2^ by 2100, with the multi‐model ensemble average temperature controlled below 2°C (O'Neill et al. 2016).

The 22 environmental variables were all included in the MaxEnt training samples for initial modeling, and the jackknife method was used to calculate the importance of each environmental variable. Subsequently, environmental variables with a low contributing rate (< 0.5%) were excluded. A pair‐wise comparison of Spearman's correlations between environmental variables from each distribution point was conducted, and pairs displaying a correlation > 0.8 were reduced with the removal of the variable of lower importance (Zhan et al. 2022; Zhao, Zhang, and Xu 2020). To avoid multi‐collinearity between environmental variables, the variance inflation factor (VIF) was employed to ensure that VIF of each variable was less than 5 (Li, Fan, and He 2020; Wang et al. 2023; Zhao et al. 2024). Consequently, five environmental variables were established as assessment variables for use in subsequent modeling (Table 1).

**TABLE 1:** Environmental variables affecting the distribution of *O*. *fragrans*.

Code	Environmental variables	Units	Contribution rate/%	Permutation importance
Bio 18	Precipitation of warmest quarter	mm	68.1	11.6
Bio 4	Temperature seasonality	°C	18.9	12.5
Bio 10	Mean temperature of warmest quarter	°C	6.2	58.3
Bio 3	Isothermality	°C	4.2	14.7
Bio 14	Precipitation of driest month	mm	2.7	3

### 2.3 Prediction of Suitable Habitats for *O. fragrans* Using the MaxEnt Model

#### 2.3.1 Construction of the MaxEnt Model

The MaxEnt model was loaded with the collected 629 *O*. *fragrans* distribution points and the five finalized environmental variables. The selected output format of the model was “Cloglog” (Phillips et al. 2017). The option for a random seed was checked, and the cross‐validation method was used to run the model 10 times (Abdelaal et al. 2019).

#### 2.3.2 Assessment of Model Accuracy

The accuracy of the modeling results was estimated from the area under the curve (AUC) of the receiver operating characteristic (ROC) plotted by the MaxEnt (Shi et al. 2024). The AUC values range from 0 to 1, with AUC values between 0.9 and 1 considered to be highly accurate. AUC values between 0.7 and 0.9 were considered average, and those between 0.5 and 0.7 suggested low accuracy (Liu et al. 2021).

To further assess the results of modeling, true skill statistics (TSS) and kappa were used for the evaluation though R package Presence Absence in R language (Freeman and Moisen 2008). The TSS values less than 0.4 were considered poor, those between 0.4 and 0.75 were considered good, and those greater than 0.75 were considered excellent (Changjun et al. 2021; Georgopoulou, Djursvoll, and Simaiakis 2016). The kappa values range from −1 to 1. When the value of kappa is more than 0.75, the model prediction is excellent (Jayasinghe and Kumar 2019; Zeiss et al. 2024).

#### 2.3.3 Classification of Suitable Habitats for *O*. *fragrans*

The modeling results were imported into ArcGIS, where the suitable habitats for *O*. *fragrans* were reclassified. Currently, the classification of suitable habitats largely relies on empirical judgment and there is no uniform standard. Habitat classification for *O*. *fragrans* used the methods of natural break (Jenks) (Zhan et al. 2022), equal method (Zhang, Sun et al. 2023), and manual division based on experience to determine appropriate thresholds (Yang et al. 2013; Zhang et al. 2019). The results were evaluated based on the current distribution. Subsequently, the habitat of *O*. *fragrans* were divided into four categories according to the probability of population survival (0–1): unsuitable areas (< 0.2), low suitability areas (0.2–0.4), moderate suitability areas (0.4–0.6), and areas of high suitability (> 0.6) (Kong et al. 2021).

## 3 Results

### 3.1 The Accuracy of the MaxEnt Model

The *O*. *fragrans* coordinates and environment parameters were imported into MaxEnt 3.4.4 to create a simulation of the potential distribution locations of *O*. *fragrans* in the context of contemporary and four future scenarios. The AUC values of the models under all climate scenarios were greater than 0.950 (Table 2), indicating high accuracy of the predictions. Furthermore, the TSS value was 0.86 and the kappa was 0.79 in the context of contemporary climate, indicating the reliability of the result.

**TABLE 2:** AUC values under various climate scenarios.

Climate scenario	Year	AUC value
Current	Current	0.957
Lowly compulsive scenario SSP1‐2.6	2021**–**2040	0.958
	2041**–**2060	0.958
	2061**–**2080	0.957
	2081**–**2100	0.958
Moderately compulsive scenario SSP2‐4.5	2021**–**2040	0.957
	2041**–**2060	0.958
	2061**–**2080	0.958
	2081**–**2100	0.958
Moderately to highly compulsive scenario SSP3‐7.0	2021**–**2040	0.957
	2041**–**2060	0.958
	2061**–**2080	0.958
	2081**–**2100	0.957
Highly compulsive scenario SSP5‐8.5	2021**–**2040	0.958
	2041**–**2060	0.957
	2061**–**2080	0.957
	2081**–**2100	0.956

Abbreviation: AUC, Area under the curve.

### 3.2 Important Environmental Variables

The five selected environmental variables were imported into the MaxEnt to predict the areas of suitable habitat for *O*. *fragrans* on the global scale. Table 1 shows that the top three contributing variables were the precipitation of the warmest quarter (Bio 18), temperature seasonality (Bio 4), and mean temperature of the warmest quarter (Bio 10). The precipitation of the warmest quarter (Bio 10), isotermality (Bio 3), and temperature seasonality (Bio 4) were the top three environmental variables of permutation importance.

**FIGURE 3:**
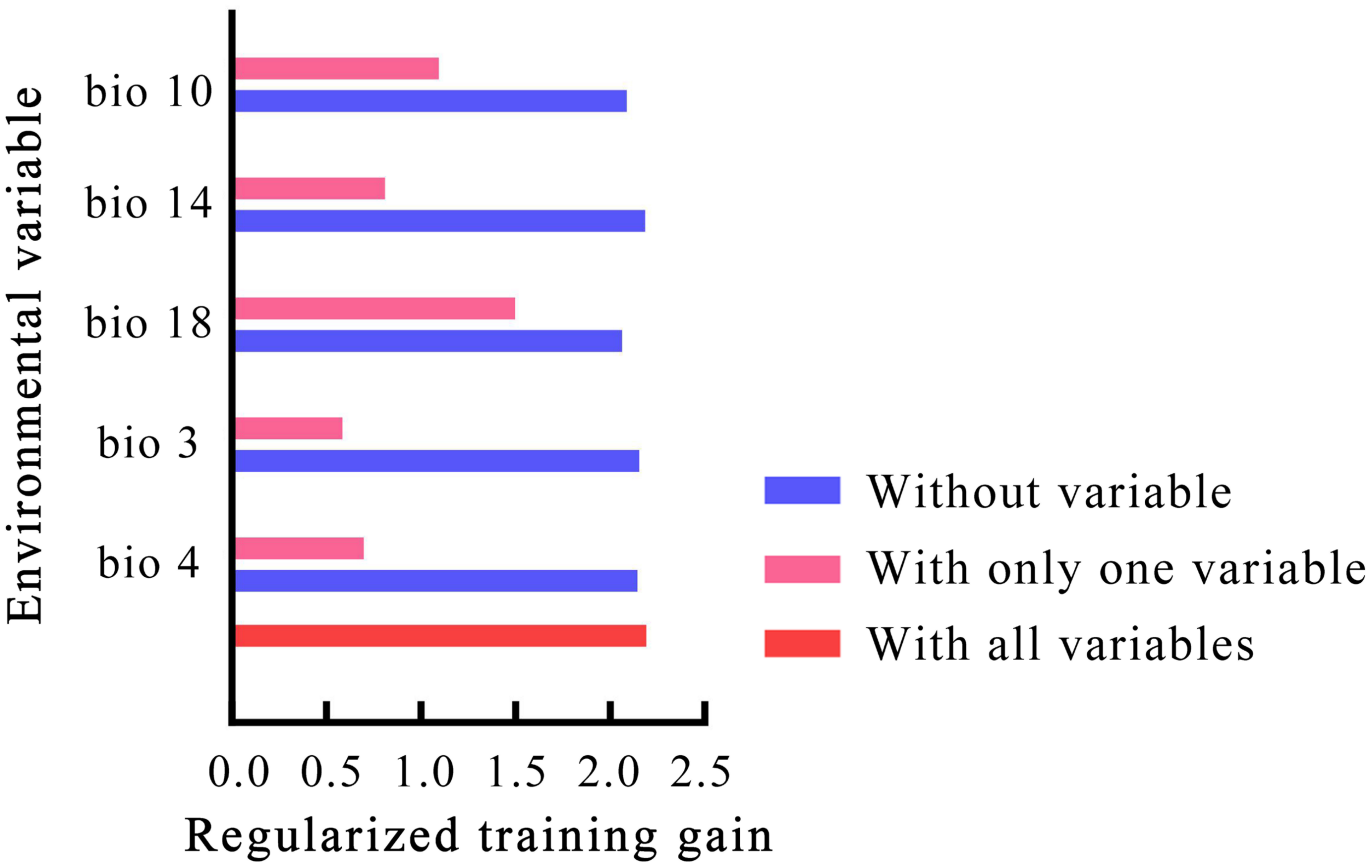
Analysis of environmental variables based on jackknife tests.

The relative importance of each environmental variable in the model construction was compared using jackknife tests with the MaxEnt (Figure 3). The pink bars represent the gain value when an environmental variable was used alone to simulate the species' distribution, and these gains positively correlate with the importance of the environmental variable for the distribution. When using a single environmental variable, the training gain values obtained with precipitation of the warmest quarter (Bio 18) and mean temperature of the warmest quarter (Bio 10) exceeded 1.0, indicating that these two variables were the most crucial environmental variables and contain the most useful information. The blue bars showed the gain values when predicting the species distribution after the omission of a particular environmental variable, which was therefore negatively correlated with the specific information contained in the environmental variable. When an environmental variable was omitted for modeling, the ones reducing gain values to a greater extent were the precipitation of the warmest quarter (Bio 18), mean temperature of the warmest quarter (Bio 10), and the temperature seasonality (Bio 4), suggesting that these variables had more unique information for predicting the distribution of *O*. *fragrans* than the others.

Response curves demonstrated the correlation between the probability of species' presence and the local environmental variables. It is generally accepted that probabilities of presence > 0.5 indicate that the range of environmental variables is suitable for a species growth. The results showed that *O*. *fragrans* grew best when precipitation of the warmest quarter (Bio 18) was above 412 mm, and the precipitation of the driest month (Bio 14) ranged from 20 to 144 mm. In addition, the isothermality (Bio 3) ranged from 25 to 44, the temperature seasonality (Bio 4) ranged from 310 to 897, and the mean temperature of the warmest quarter (Bio 10) was between 23°C and 29°C, which were indicated as the most suitable temperature conditions for *O*. *fragrans* growth (Figure S1).

### 3.3 Potential Suitable Habitats for *O. fragrans* Based on Current Climatic Conditions

**FIGURE 4:**
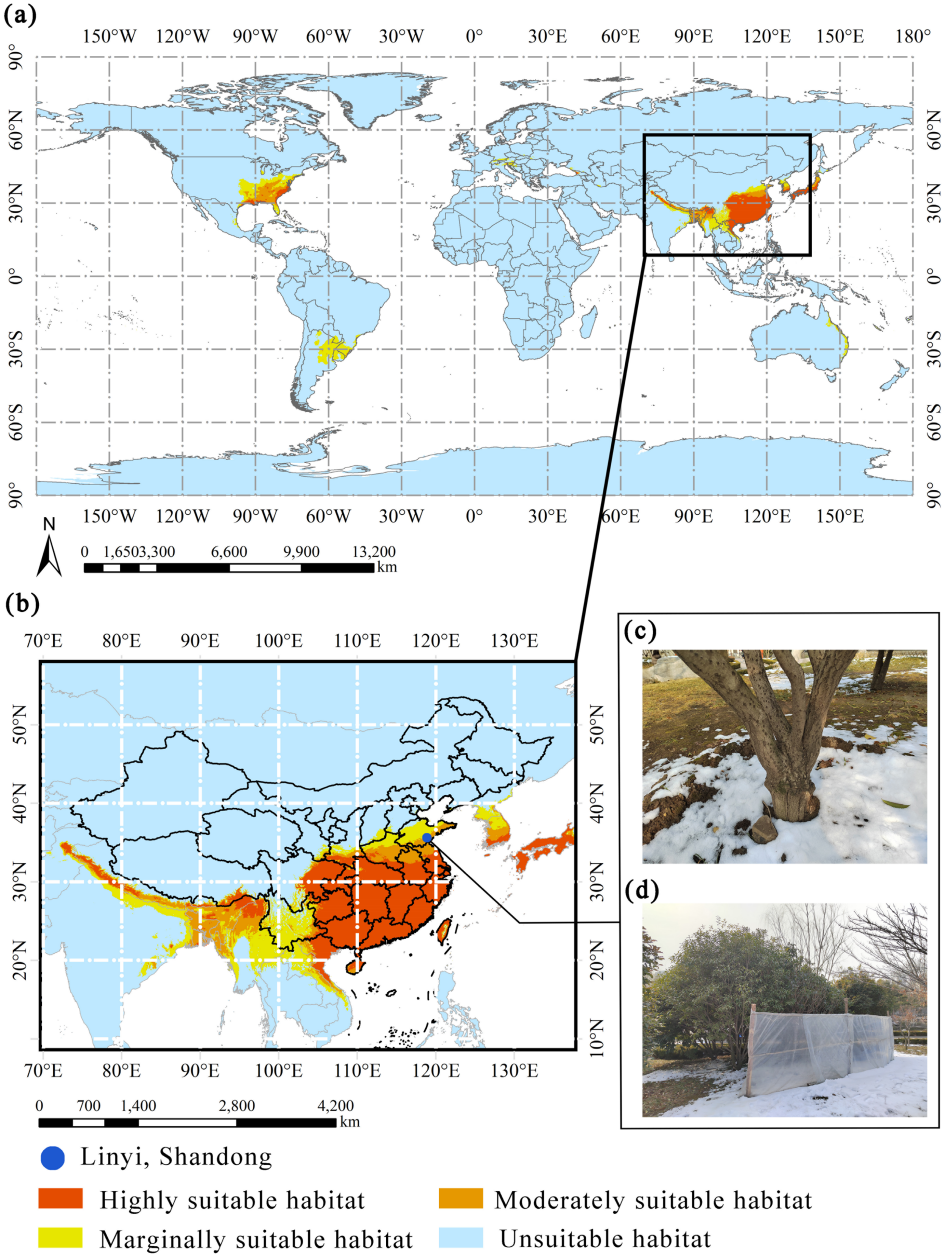
The distribution sites and potential distribution of *O*. *fragrans* under current climatic conditions (a) in the world and (b) in China. (c) The grafted *O*. *fragrans*. (d) The windbreaks set up in Linyi, Shandong, to help *O*. *fragrans* overwinter.

According to the findings, *O*. *fragrans* can be distributed between 15° N–50° N and 15° S–40° S under current climatic conditions. The suitable habitats for *O*. *fragrans* were mostly located in the eastern coastal areas of the continents at medium and low latitudes (Figure 4a). The image pixels of *O*. *fragrans* in each class of habitat were counted by ArcGIS, and the analysis showed that under contemporary climatic conditions, the total area of suitable habitats globally was 802.76 × 10^4^ km^2^, which includes 265.03 × 10^4^ km^2^ for high suitability areas, 171.33 × 10^4^ km^2^ for moderate suitability areas, and 366.40 × 10^4^ km^2^ for low suitability areas. The largest suitable area for *O*. *fragrans* was in Asia, followed by North and South America, with smaller suitable areas in Oceania and Europe (Figure 4a). The suitable area of each continent accounted for 56.57%, 25.67%, 13.14%, 2.69%, and 1.84% of the total habitat. There were few suitable areas for *O*. *fragrans* in Africa.

In Asia, the suitable habitats for *O*. *fragrans* mainly included China, Japan, Vietnam, Myanmar, and the low altitude areas on the southern slopes of the Himalayas. In North America, the mid‐latitude Atlantic coast was suitable for *O*. *fragrans*, transitioning from highly suitable areas along the coast to low suitability areas inland. The Pampas grasslands along the Atlantic coast of South America was the marginally suitable areas for *O*. *fragrans*. In Oceania, the narrow strip along the east coast of Australia was mostly low suitability areas. In Europe, scattered suitable areas were found in the Mediterranean region south of the Alps and along the Black Sea coast (Figure 4a).

In China, the highly suitable areas for *O*. *fragrans* were mainly concentrated south of the Qinling Mountains‐Huaihe River Line, including east Sichuan, Shanghai, Zhejiang, Jiangxi, Hubei, Hunan, Chongqing, Guizhou, Fujian, Guangzhou, Guangxi, and Taiwan, as well as the southern part of Jiangsu, Anhui, and Shaanxi. Areas of moderate and low suitability were distributed in Shandong, Henan, Yunnan, and Hainan (Figure 4b).

### 3.4 Changes of Suitable Habitats of O. Fragrans Under Future Scenarios

**FIGURE 5:**
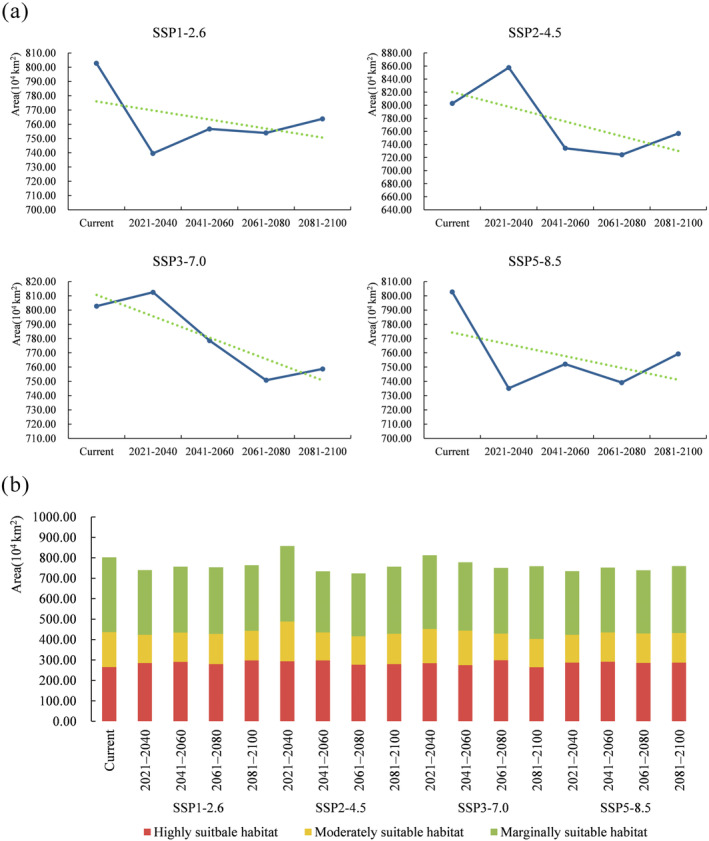
The change of suitable habitats area of *O*. *fragrans* under future climate scenarios. (a) Area line chart of total suitable habitats of *O*. *fragrans* under four climate scenarios. The dotted line represents the trend of change. (b) Area of each suitability class under different climate scenarios.

Comparing the predicted outcomes of *O*. *fragrans* suitable areas in future climate scenarios and the current potential distribution, we gained a general understanding of the modifications in the species' suitable habitat. Compared with the contemporary area of potential habitat, under the SSP1‐2.6 scenario, the total suitable area for *O*. *fragrans* in all four periods was less than that in the contemporary (Figure 5a). Between 2021 and 2040, there was a significant decrease in the total suitable area, representing 739.53 × 10^4^ km^2^. The scenario resulted in a reduction in the area of moderately and marginally suitable habitat, while the area of high suitability increased by 12.38% during 2081–2100, which was the largest increase (Figure 5b). Similarly, under the SSP5‐8.5 scenario, the total suitable area was less than the contemporary one in all four periods. The largest decrease of area was found in 2021–2040 at 735.13 × 10^4^ km^2^, which was 8.42% less than the area of contemporary total suitable habitat (Figure 5a). With both the area of moderate and low suitable habitat decreased, the area of the high suitability increased between 2021 and 2100 and the greatest increase in the area of highly suitable habitat was observed in 2041–2060, with an increase of 9.87% compared to contemporary (Figure 5b).

The total habitat area of *O*. *fragrans* under SSP2‐4.5 and SSP3‐7.0 scenarios showed a decreasing trend after an increase (Figure 5a). Between 2021 and 2040, the total suitable area under the SSP2‐4.5 scenario was 857.58 × 10^4^ km^2^, an increase of 6.83%, while under the SSP3‐7.0 scenario, the total suitable area was 812.50 × 10^4^ km^2^, an increase of 1.21%. From 2041 to 2100, the total suitable area under both scenarios was reduced compared to the contemporary total suitable area. Under all scenarios, the SSP2‐4.5 scenario showed the largest reduction in the total suitable area of 724.21 × 10^4^ km^2^ in 2061–2080, which was 9.78% less than the contemporary (Figure 5a). In summary, under future climate scenarios with increasing radiative forcing, the global suitable area for *O*. *fragrans* showed an overall tendency to decrease (Figure 5).

**FIGURE 6:**
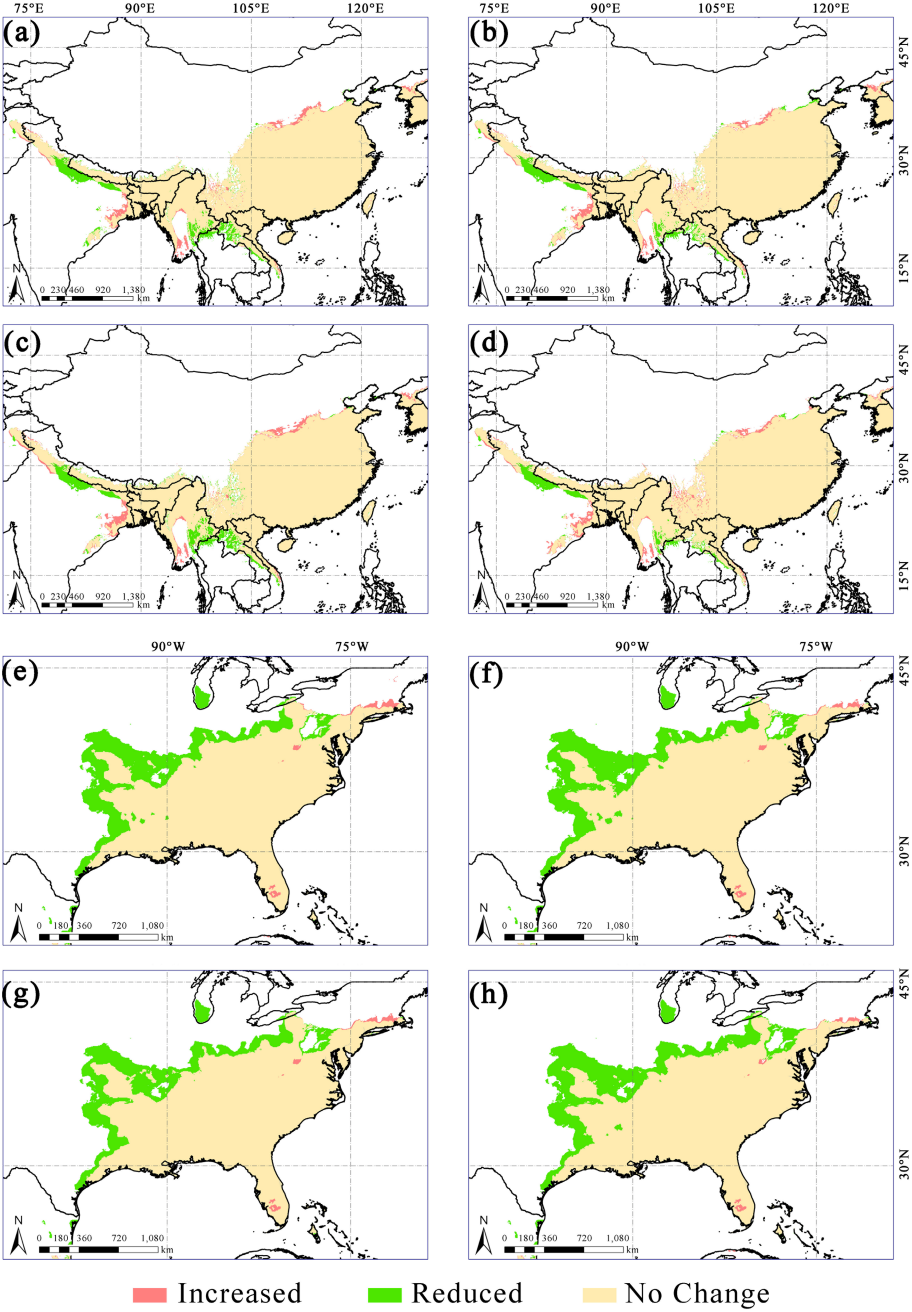
Changes in main habitats of *O*. *fragrans* under future scenarios. (a) The changes in Asia under SSP1‐2.6, 2021–2040 and (b) 2041–2060. (c) The changes in Asia under SSP5‐8.5, 2021–2040 and (d) 2041–2060. (e) The changes in North America under SSP1‐2.6, 2021–2040 and (f) 2041–2060. (g) The changes in North America under SSP5‐8.5, 2021–2040 and (h) 2041–2060.

Taking the strongest (SSP5‐8.5) and weakest (SSP1‐2.6) forced scenarios as examples, comparing the contemporary map of suitable areas with those predicted under the different climate scenarios in the future, it was found that the areas where suitable areas decreased were mostly in the interior of the Americas and parts of the Himalayas, while suitable areas in Asia were seen to expand from mid‐ to high‐latitude regions (Figure 6).

### 3.5 Shifts of the Suitable Habitat Distribution Center

**FIGURE 7:**
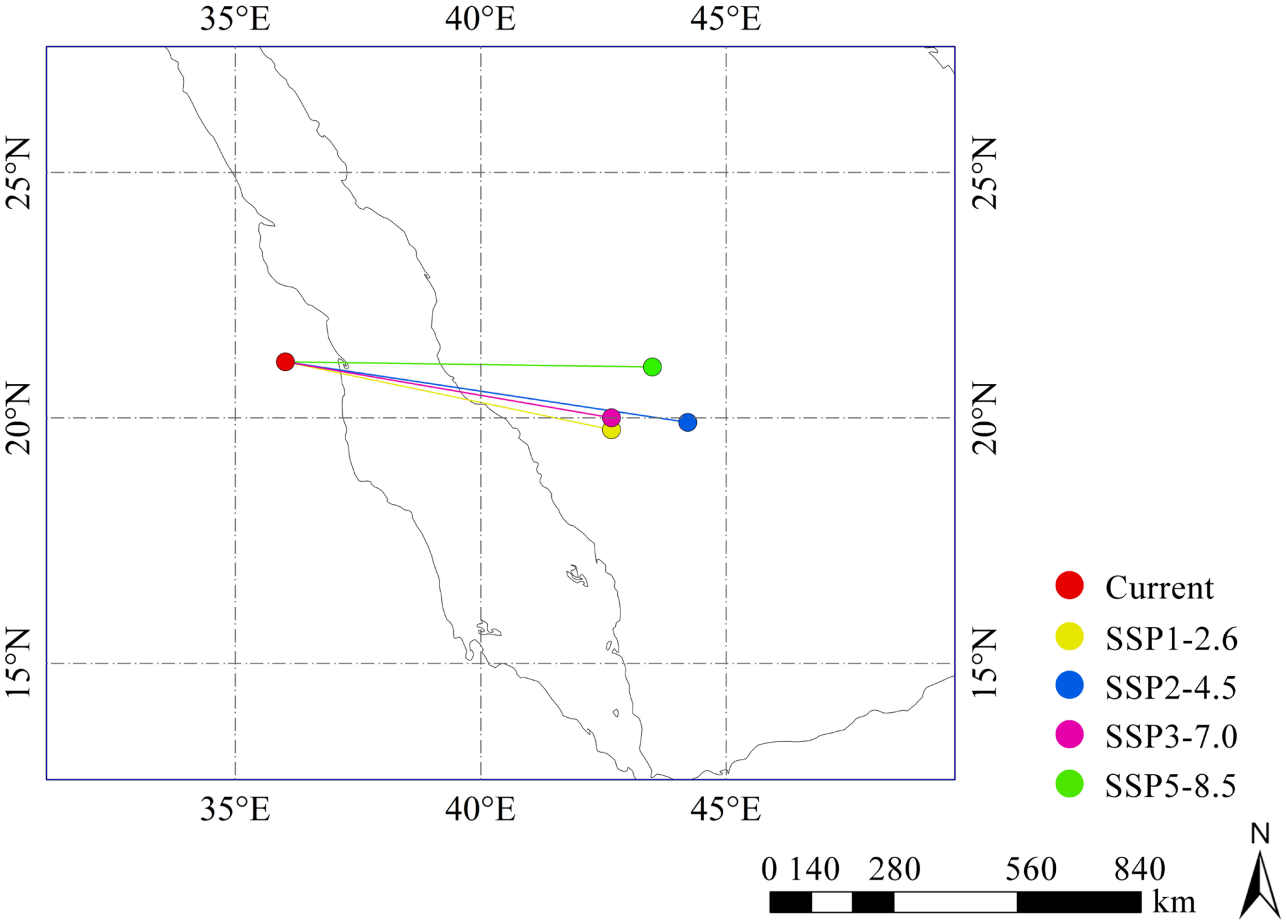
Shifts in the centroid of global *O*. *fragrans* habitat under different scenarios in 2081–2100.

The centroid coordinate of the current habitat of *O*. *fragrans* all over the world was 21°8′36″ N, 36°1′16″ E. By 2081–2100, the centroid of SSP1‐2.6 was projected to be located at 19°45′43″ N, 42°39′59″ E, and the centroid of SSP2‐4.5 was located at 19°54′45″ N, 44°13′13″ E. The centroid of the global suitable area for *O*. *fragrans* under SSP3‐7.0 and SSP5‐8.5 was 20°0′21″ N, 42°40′2″ E and 21°2′19″ N, 43°29′54″ E, respectively (Figure 7). In summary, the centroid of global suitable areas showed a southeastern shift under all four scenarios. The furthest distance from the centroid of contemporary climatic conditions was the centroid of SSP2‐4.5, which was displaced 864.56 km to the southeast.

## 4 Discussion

In this study, we found that the suitable habitat for *O*. *fragrans* is concentrated in the eastern coastal areas of the continents at medium and low latitudes, and the suitable area will decrease in the future. Methodologically similar predictive studies of *O*. *fragrans* have been reported, but these were geographically limited in scope (Kong et al. 2021; Wu et al. 2022). The distribution range of *O*. *fragrans* in China in this study was wider than previous studies, which was influenced by the input data and parameter settings. Here, occurrence records from 629 valid coordinates were utilized to provide a global scale perspective, which contains 460 coordinates in China. In addition, this study utilized the latest models of climate change from CMIP6 to predict changes in environmental factors at a local scale and their potential effects on *O*. *fragrans* distribution. Compared with CMIP5, the data of CMIP6 had a higher resolution and could more closely match the trends in climate change in recent years.

The accuracy of the MaxEnt model predictions was primarily influenced by the input data and parameter settings. All AUC values in this paper were tested to be above 0.9, while the TSS and kappa values for current climatic conditions surpassed 0.75, indicating the high accuracy of prediction models.

### 4.1 Impact of Environmental Variables

Climatic factors have a significant impact on the individual development and population dynamics of plants. Temperature and precipitation are two crucial environmental factors that greatly affect the habitable zone of plants. This study used the jackknife method to identify key environmental factors that have an impact on the distribution of *O*. *fragrans*. It was found that the precipitation of the warmest quarter (Bio 18) and the mean temperature of the warmest quarter (Bio 10) had the most significant impact on the distribution of *O*. *fragrans*. The MaxEnt model predicted that optimal suitable habitats for *O*. *fragrans* had precipitation in the warmest season of at least 412 mm and a mean temperature in the warmest quarter between 23°C and 29°C. This is consistent with the environmental conditions in Suzhou, Jangsu, which is one of the five major *O*. *fragrans*‐producing areas in China and over the last 10 years (2013–2022) has shown 565 mm precipitation in the warmest season and an average temperature in the warmest season of 28°C (data from the Bureau of Statistics; www.tjj.suzhou.gov.cn).

*O*. *fragrans* thrives best in warm and moist environments and has high water requirements (Mu et al. 2023). Earlier studies suggested that *Osmanthus* belonged to the pantropical distribution type, with intermittent distribution of subtypes in tropical Asia, Oceania, and Central and South America (Xiang and Liu 2008). The predicted results showed that the potential suitable areas for *O*. *fragrans* globally were mainly concentrated in the eastern coastal areas of the continents in mid and high latitudes, which is consistent with previous analyses. These areas have subtropical monsoon and humid climate with abundant rain in summer and a warm and humid winter. This climatic feature is consistent with the dominant optimal precipitation (Bio 18) and temperature (Bio 10) factors shown by *O*. *fragrans*. The geographic and climatic conditions in the predicted potential habitats for *O*. *fragrans* reflected its preference for warm and humid growing conditions.

### 4.2 Changes of Appropriate Potential Habitats

From the results, it was clear that the availability of suitable habitats for *O*. *fragrans* will decrease in the future. Based on current climatic conditions, *O*. *fragrans* was distributed between 15° N–50° N and 15° S–40° S, with a clear distribution characteristic that mostly concentrated in the eastern coastal regions of continents with subtropical monsoons and humid climate types. In the context of global warming, temperatures in areas of higher latitudes will become more suitable for the survival of *O*. *fragrans*. However, precipitation is decreasing in subtropical regions around the world, and this trend change is expected to continue (Anadon, Sala, and Maestre 2014; Li, Deng et al. 2022). The current analysis indicates that precipitation is an important climatic factor currently determining the distribution of *O*. *fragrans*, and that the global decrease of precipitation in subtropical regions is predicted to result in further reduction of suitable habitats. The southeastern shift of the *O*. *fragrans* centroid was thought to be mainly due to the reduction of habitats in Americas. The results of predictive modeling showed that the areas mainly affected were located in the interior of Americas. In Asia, the reduction was predicted to occur in the southern Himalayan regions of northern India, but with a slight trend of expansion along the eastern coast of India, which is expected to display a more tropical monsoon climate with a higher level of precipitation during the warmest season, which would thus be more favorable for *O*. *fragrans* growth and survivability (Li, Shao, and Jiang 2022). In agreement with a previous study (Wu et al. 2022), our data also predicted that suitable habitats in China will change from medium to high latitudes under the future climate scenario.

### 4.3 Implications for Promotion and Conservation

It is reported that the global warming is affecting the distribution of species (Bellard et al. 2012). However, some species show a greater capacity to physiologically adapt to these changes (Hu et al. 2015). Currently, research into methods which facilitate plant adaption to climate change is receiving more and more attention. *O*. *fragrans* is an endemic species in China, which has a broad application prospect in landscaping. Under contemporary climate scenarios, the Henan and Shandong provinces of China are moderate or marginally suitable habitats for *O*. *fragrans*, and according to field research in these provinces, *O*. *fragrans* planted in the open ground is mostly grafted seedlings. Grafting is a common method of artificial propagation, and in recent years, it has been widely used in research concerned with plant resistance (Koepke and Dhingra 2013). The rootstocks commonly used for grafting *O*. *fragrans* include *Ligustrum lucidum* and *Chionanthus retusus*. Because *C*. *retusus* displays better drought and cold tolerance, this species is more often used as the rootstock in the north of China. Differences in resistance among different varieties of *O*. *fragrans* and microclimate environments also affect the survival and growth of species in areas of moderate and low suitability. In Linyi, Shandong Province, for example, windbreaks are often erected in winter to create a suitable microclimate for *O*. *fragrans* planted in the open ground. Therefore, in preparation for future climate changes, further research into *O*. *fragrans* grafting and other cultivation techniques is needed. In addition, a comprehensive investigation and conservation of wild *O*. *fragrans* genetic resources would be helpful for the breeding of drought‐resistant varieties to meet the market demands.

## 5 Conclusion

This study was based on the hypothesis that the distribution of species was primarily determined by climate and topography. Using the MaxEnt model, the study forecasted the possible geographical distribution of *O*. *fragrans* under present and future climate conditions. According to the predictions of MaxEnt, under future climate scenarios, the total potential suitable area of *O*. *fragrans* globally showed a decreasing trend. The geographic and climatic conditions in the predicted potential habitats for *O*. *fragrans* showed that the precipitation of warmest quarter (Bio 18) and mean temperature of the warmest quarter (Bio 10) were the most important environmental factors affecting the growth of *O*. *fragrans*, implying *O*. *fragrans* preference for warm and humid growing conditions. Future research into *O*. *fragrans* distribution should include more field surveys and incorporate more biotic and abiotic data to improve the accuracy of distribution predictions. Additionally, a closer inspection of the environmental requirements of *O*. *fragrans* is required. Combined with the biological habitats of *O*. *fragrans*, the important environmental factors derived from this study can provide a reference basis for the breeding for stress resistance. It is possible to plan for the introduction, cultivation, and promotion of *O*. *fragrans* by taking into account future changes in the distribution of suitable habitats.

## Author Contributions

**Yuanzheng Yue:** conceptualization (lead), funding acquisition (equal), investigation (equal), project administration (equal), validation (equal), writing – review and editing (equal). **Yingyu Huang:** data curation (lead), formal analysis (lead), methodology (lead), visualization (lead), writing – original draft (lead). **Wei Liu:** investigation (equal), software (lead). **Xiulian Yang:** funding acquisition (equal), project administration (equal), resources (equal), validation (equal). **Lianggui Wang:** funding acquisition (equal), project administration (equal), resources (equal).

## Conflicts of Interest

The authors declare no conflicts of interest.

## Supporting information


**Figure S1.** Response curves between probability of presence and environmental variables.

## Data Availability

The data supporting the findings of this study were obtained from The Global Biodiversity Information Facility (GBIF, http://www.gbif.org), the China Virtual Herbarium (http://www.cvh.ac.cn), Taiwan Biodiversity Network (http://tbn.org.tw), Atlas of Living Australia (https://www.ala.org.au), and iDigBio (http://idigbio.org). The bioclimatic variables obtained from the WorldClim (http://www.worldclim.org).

## References

[ece370435-bib-0001] Abdelaal, M. , M. Fois , G. Fenu , and G. Bacchetta . 2019. “Using MaxEnt Modeling to Predict the Potential Distribution of the Endemic Plant *Rosa arabica* crép. In Egypt.” Ecological Informatics 50: 68–75. 10.1016/j.ecoinf.2019.01.003.

[ece370435-bib-0002] Adhikari, B. , S. C. Subedi , S. Bhandari , K. Baral , S. Lamichhane , and T. Maraseni . 2023. “Climate‐Driven Decline in the Habitat of the Endemic Spiny Babbler (*Turdoides nipalensis*).” Ecosphere 14: e4584. 10.1002/ecs2.4584.

[ece370435-bib-0003] Ahmed, S. E. , G. Mcinerny , K. O'Hara , et al. 2015. “Scientists and Software – Surveying the Species Distribution Modelling Community.” Diversity and Distributions 21: 258–267. 10.1111/ddi.12305.

[ece370435-bib-0004] Aiello‐Lammens, M. E. , R. A. Boria , A. Radosavljević , B. Vilela , and R. P. Anderson . 2015. “spThin: An R Package for Spatial Thinning of Species Occurrence Records for Use in Ecological Niche Models.” Ecography 38: 541–545. 10.1111/ecog.01132.

[ece370435-bib-0005] Anadon, J. D. , O. E. Sala , and F. T. Maestre . 2014. “Climate Change Will Increase Savannas at the Expense of Forests and Treeless Vegetation in Tropical and Subtropical Americas.” Journal of Ecology 102: 1363–1373. 10.1111/1365-2745.12325.

[ece370435-bib-0006] Bellard, C. , C. Bertelsmeier , P. Leadley , W. Thuiller , and F. Courchamp . 2012. “Impacts of Climate Change on the Future of Biodiversity.” Ecology Letters 15: 365–377. 10.1111/j.1461-0248.2011.01736.x. 22257223 PMC3880584

[ece370435-bib-0007] Changjun, G. , T. Yanli , L. Linshan , et al. 2021. “Predicting the Potential Global Distribution of *Ageratina adenophora* Under Current and Future Climate Change Scenarios.” Ecology and Evolution 11: 12092–12113. 10.1002/ece3.7974. 34522363 PMC8427655

[ece370435-bib-0008] Chen, I. C. , J. K. Hill , R. Ohlemuller , D. B. Roy , and C. D. Thomas . 2011. “Rapid Range Shifts of Species Associated With High Levels of Climate Warming.” Science 333: 1024–1026. 10.1126/science.1206432. 21852500

[ece370435-bib-0009] Chen, T. , and X. Gong . 2022. “Global Research Trend Analysis of *Osmanthus fragrans* Based on Bibliometrix.” Mobile Information Systems 2022: 4091962. 10.1155/2022/4091962.

[ece370435-bib-0010] Cuena‐Lombraña, A. , M. Fois , G. Fenu , D. Cogoni , and G. Bacchetta . 2018. “The Impact of Climatic Variations on the Reproductive Success of *Ggentiana lutea* L. in a Mediterranean Mountain Area.” International Journal of Biometeorology 62: 1283–1295. 10.1007/s00484-018-1533-3. 29602965

[ece370435-bib-0011] Dunn, W. C. , and B. T. Milne . 2014. “Implications of Climatic Heterogeneity for Conservation of the Lesser Prairie‐Chicken (*Tympanuchus pallidicinctus*).” Ecosphere 5: 1–17. 10.1890/ES13-00333.1.

[ece370435-bib-0012] Freeman, E. A. , and G. G. Moisen . 2008. “A Comparison of the Performance of Threshold Criteria for Binary Classification in Terms of Predicted Prevalence and Kappa.” Ecological Modelling 217: 48–58. 10.1016/j.ecolmodel.2008.05.015.

[ece370435-bib-0013] Georgopoulou, E. , P. Djursvoll , and S. M. Simaiakis . 2016. “Predicting Species Richness and Distribution Ranges of Centipedes at the Northern Edge of Europe.” Acta Oecoligica 74: 1–10. 10.1016/j.actao.2016.03.006.

[ece370435-bib-0014] Han, Y. , H. Wang , X. Wang , et al. 2019. “Mechanism of Floral Scent Production in *Osmanthus fragrans* and the Production and Regulation of Its Key Floral Constituents, β‐Ionone and Linalool.” Horticulture Research 6: 106. 10.1038/s41438-019-0189-4. 31645961 PMC6804851

[ece370435-bib-0015] Hu, S. , S. Wu , Y. Wang , H. Zhao , and Y. Zhang . 2014. “Genetic Diversity and Genetic Structure of Different Types of Natural Populations in *Osmanthus fragrans* Lour. And the Relationships With Sex Ratio, Population Structure, and Geographic Isolation.” Scientific World Journal 2014: 817080. 10.1155/2014/817080. 25436228 PMC4243123

[ece370435-bib-0016] Hu, X. , Y. Jin , X. Wang , J. Mao , and Y. Li . 2015. “Predicting Impacts of Future Climate Change on the Distribution of the Widespread Conifer *Platycladus orientalis* .” PLoS One 10: e0132326. 10.1371/journal.pone.0132326. 26132163 PMC4488561

[ece370435-bib-0017] Hung, C. , Y. Tsai , and K. Li . 2012. “Phenolic Antioxidants Isolated From the Flowers of *Osmanthus fragrans* .” Molecules 17: 10724–10737. 10.3390/molecules170910724. 22960867 PMC6268160

[ece370435-bib-0018] Jayasinghe, S. L. , and L. Kumar . 2019. “Modeling the Climate Suitability of Tea [*Camellia Sinensis* (L.) O. Kuntze] in Sri Lanka in Response to Current and Future Climate Change Scenarios.” Agricultural and Forest Meteorology 272: 102–117. 10.1016/j.agrformet.2019.03.025.

[ece370435-bib-0019] Jiao, X. , M. Long , J. Li , Q. Yang , and Z. Liu . 2023. “Reconstructing the Invasive History and Potential Distribution Prediction of *Amaranthus palmeri* in China.” Agronomy 13: 2498. 10.3390/agronomy13102498.

[ece370435-bib-0020] Koepke, T. , and A. Dhingra . 2013. “Rootstock Scion Somatogenetic Interactions in Perennial Composite Plants.” Plant Cell Reports 32: 1321–1337. 10.1007/s00299-013-1471-9. 23793453 PMC4244527

[ece370435-bib-0021] Kong, F. , L. Tang , H. He , F. Yang , J. Tao , and W. Wang . 2021. “Assessing the Impact of Climate Change on the Distribution of *Osmanthus fragrans* Using Maxent.” Environmental Science and Pollution Research 28: 34655–34663. 10.1007/s11356-021-13121-3. 33655479

[ece370435-bib-0022] Li, H. Q. , X. H. Liu , J. H. Wang , L. G. Xing , and Y. Y. Fu . 2019. “Maxent Modelling for Predicting Climate Change Effects on the Potential Planting Area of Tuber Mustard in China.” Journal of Agricultural Science 157: 375–381. 10.1017/S0021859619000686.

[ece370435-bib-0023] Li, J. , G. Fan , and Y. He . 2020. “Predicting the Current and Future Distribution of Three *Coptis* Herbs in China Under Climate Change Conditions, Using the MaxEnt Model and Chemical Analysis.” Science of the Total Environment 698: 134141. 10.1016/j.scitotenv.2019.134141. 31505366

[ece370435-bib-0024] Li, Q. , Y. Qi , Q. Wang , and D. Wang . 2022. “Prediction of the Potential Distribution of *Vaccinium uliginosum* in China Based on the Maxent Niche Model.” Horticulturae 8: 1202. 10.3390/horticulturae8121202.

[ece370435-bib-0025] Li, Y. , Y. Deng , H. Cheung , W. Zhou , S. Yang , and H. Zhang . 2022. “Amplifying Subtropical Hydrological Transition Over China in Early Summer Tied to Weakened Mid‐Latitude Synoptic Disturbances.” npj Climate and Atmospheric Science 5: 40. 10.1038/s41612-022-00259-1.

[ece370435-bib-0026] Li, Y. , W. Shao , and J. Jiang . 2022. “Predicting the Potential Global Distribution of *Sapindus Mukorossi* Under Climate Change Based on Maxent Modelling.” Environmental Science and Pollution Research 29: 21751–21768. 10.1007/s11356-021-17294-9. 34773237

[ece370435-bib-0027] Liu, L. , L. Guan , H. Zhao , et al. 2021. “Modeling Habitat Suitability of *Houttuynia cordata* Thunb (Ceercao) Using MaxEnt Under Climate Change in China.” Ecological Informatics 63: 101324. 10.1016/j.ecoinf.2021.101324.

[ece370435-bib-0028] Mccormack, J. E. , A. J. Zellmer , and L. L. Knowles . 2010. “Does Niche Divergence Accompany Allopatric Divergence in Aphelocoma Jays as Predicted Under Ecological Speciation?: Insights From Tests With Niche Models.” Evolution 64: 1231–1244. 10.1111/j.1558-5646.2009.00900.x. 19922442

[ece370435-bib-0029] Mu, H. , W. Wang , L. Fan , C. Wu , X. Guo , and T. Sun . 2023. “Effects of Piriformospora Indica on Growth and Drought Resistance in *Osmanthus fragrans* Under Water Deficit Stress.” Journal of Nanjing Forestry University (Natural Sciences Edition) 47: 101–106. 10.12302/j.issn.1000-2006.202203014.

[ece370435-bib-0030] O'Neill, B. C. , C. Tebaldi , D. P. van Vuuren , et al. 2016. “The Scenario Model Intercomparison Project (ScenarioMIP) for CMIP6.” Geoscientific Model Development 9: 3461–3482. 10.5194/gmd-9-3461-2016.

[ece370435-bib-0031] Parveen, S. , S. Kaur , R. Baishya , and S. Goel . 2022. “Predicting the Potential Suitable Habitats of Genus *Nymphaea* in India Using MaxEnt Modeling.” Environmental Monitoring and Assessment 194: 853. 10.1007/s10661-022-10524-8. 36203117

[ece370435-bib-0032] Peñuelas, J. , J. Sardans , M. Estiarte , et al. 2013. “Evidence of Current Impact of Climate Change on Life: A Walk From Genes to the Biosphere.” Global Change Biology 19: 2303–2338. 10.1111/gcb.12143. 23505157

[ece370435-bib-0033] Phillips, S. J. , R. P. Anderson , M. Dudik , R. E. Schapire , and M. E. Blair . 2017. “Opening the Black Box: An Open‐Source Release of Maxent.” Ecography 40: 887–893. 10.1111/ecog.03049.

[ece370435-bib-0034] Shi, J. , M. Xia , G. He , et al. 2024. “Predicting *Qercus gilva* Distribution Dynamics and Its Response to Climate Change Induced by GHGs Emission Through MaxEnt Modeling.” Journal of Environmental Management 357: 120841. 10.1016/j.jenvman.2024.120841. 38581898

[ece370435-bib-0035] Wang, Y. , L. Xie , X. Zhou , R. Chen , G. Zhao , and F. Zhang . 2023. “Prediction of the Potentially Suitable Areas of *Leonurus japonicus* in China Based on Future Climate Change Using the Optimized MaxEnt Model.” Ecology and Evolution 13: e10597. 10.1002/ece3.10597. 37869439 PMC10585429

[ece370435-bib-0036] Wu, T. , R. Yu , Y. Lu , et al. 2021. “BCC‐CSM2‐HR: A High‐Resolution Version of the Beijing Climate Center Climate System Model.” Geoscientific Model Development 14: 2977–3006. 10.5194/gmd-14-2977-2021.

[ece370435-bib-0037] Wu, Y. , M. Zhang , Y. Yang , Z. Lv , X. Zhang , and L. Wang . 2022. “Effects of Climate Changes on the Distribution of *Osmanthus fragrans* .” Journal of Northwest Forestry University 37: 129–134. 10.3969/j.issn.1001-7461.2022.04.17.

[ece370435-bib-0038] Xiang, Q. , and Y. Liu . 2008. An Illustrated Monograph of the Sweet Osmanthus Cultivars in China. Hangzhou: Zhejiang Science and Technology Press.

[ece370435-bib-0039] Xu, N. , F. Meng , G. Zhou , Y. Li , B. Wang , and H. Lu . 2020. “Assessing the Suitable Cultivation Areas for *Scutellaria baicalensis* in China Using the Maxent Model and Multiple Linear Regression.” Biochemical Systematics and Ecology 90: 104052. 10.1016/j.bse.2020.104052.

[ece370435-bib-0040] Yang, X. , S. P. S. Kushwaha , S. Saran , J. Xu , and P. S. Roy . 2013. “Maxent Modeling for Predicting the Potential Distribution of Medicinal Plant, *Justicia adhatoda* L. in Lesser Himalayan Foothills.” Ecological Engineering 51: 83–87. 10.1016/j.ecoleng.2012.12.004.

[ece370435-bib-0041] Yi, Y. , X. Cheng , Z. Yang , and S. Zhang . 2016. “Maxent Modeling for Predicting the Potential Distribution of Endangered Medicinal Plant (*H. riparia* Lour) in Yunnan, China.” Ecological Engineering 92: 260–269. 10.1016/j.ecoleng.2016.04.010.

[ece370435-bib-0042] Zeiss, R. , M. J. I. Briones , J. Mathieu , et al. 2024. “Effects of Climate on the Distribution and Conservation of Commonly Observed European Earthworms.” Conservation Biology 38: e14187. 10.1111/cobi.14187. 37768192

[ece370435-bib-0043] Zhan, P. , F. Wang , P. Xia , et al. 2022. “Assessment of Suitable Cultivation Region for *Panax notoginseng* Under Different Climatic Conditions Using MaxEnt Model and High‐Performance Liquid Chromatography in China.” Industrial Crops and Products 176: 114416. 10.1016/j.indcrop.2021.114416.

[ece370435-bib-0044] Zhang, H. , X. Sun , G. Zhang , et al. 2023. “Potential Global Distribution of the Habitat of Endangered *Gentiana Rhodantha Franch*: Predictions Based on MaxEnt Ecological Niche Modeling.” Sustainability 15: 631. 10.3390/su15010631.

[ece370435-bib-0045] Zhang, J. , F. Jiang , G. Li , et al. 2019. “Maxent Modeling for Predicting the Spatial Distribution of Three Raptors in the Sanjiangyuan National Park, China.” Ecology and Evolution 9: 6643–6654. 10.1002/ece3.5243. 31236249 PMC6580265

[ece370435-bib-0046] Zhang, Q. , X. Shen , X. Jiang , T. Fan , X. Liang , and W. Yan . 2023. “MaxEnt Modeling for Predicting Suitable Habitat for Endangered Tree *Keteleeria Davidiana* (Pinaceae) in China.” Forests 14: 394. 10.3390/f14020394.

[ece370435-bib-0047] Zhang, Z. , D. Fan , S. Guo , D. Li , and Z. Zhang . 2011. “Development of 29 Microsatellite Markers for *Osmanthus fragrans* (Oleaceae), a Traditional Fragrant Flowering Tree of China.” American Journal of Botany 98: e356–e359. 10.3732/ajb.1100241. 22114222

[ece370435-bib-0048] Zhao, H. , H. Zhang , and C. Xu . 2020. “Study on *Taiwania Cryptomerioides* Under Climate Change: MaxEnt Modeling for Predicting the Potential Geographical Distribution.” Global Ecology and Conservation 24: e01313. 10.1016/j.gecco.2020.e01313.

[ece370435-bib-0049] Zhao, Z. , X. Feng , Y. Wang , Z. Zhou , and Y. Zhang . 2024. “Potential Suitability Areas of *Sitobion miscanthi* in China Based on the MaxEnt Model: Implications for Management.” Crop Protection 183: 106755. 10.1016/j.cropro.2024.106755.

[ece370435-bib-0050] Zheng, Z. , Q. Xu , J. Tang , et al. 2023. “Genome‐Wide Analysis of TCP Gene Family in *Osmanthus fragrans* Reveals a Class I Gene *Of* *TCP13* Modulate Leaf Morphology.” Ornamental Plant Research 3. 10.48130/OPR-2023-0015.

