# Supplementary figures and images for: Predicting the Global Potential Suitable Areas of Sweet Osmanthus (Osmanthus fragrans) Under Current and Future Climate Scenarios
Source: Ecol Evol. 2024 Nov 5;14(11):e70435. doi: 10.1002/ece3.70435 (PMC11537704; doi:10.1002/ece3.70435)

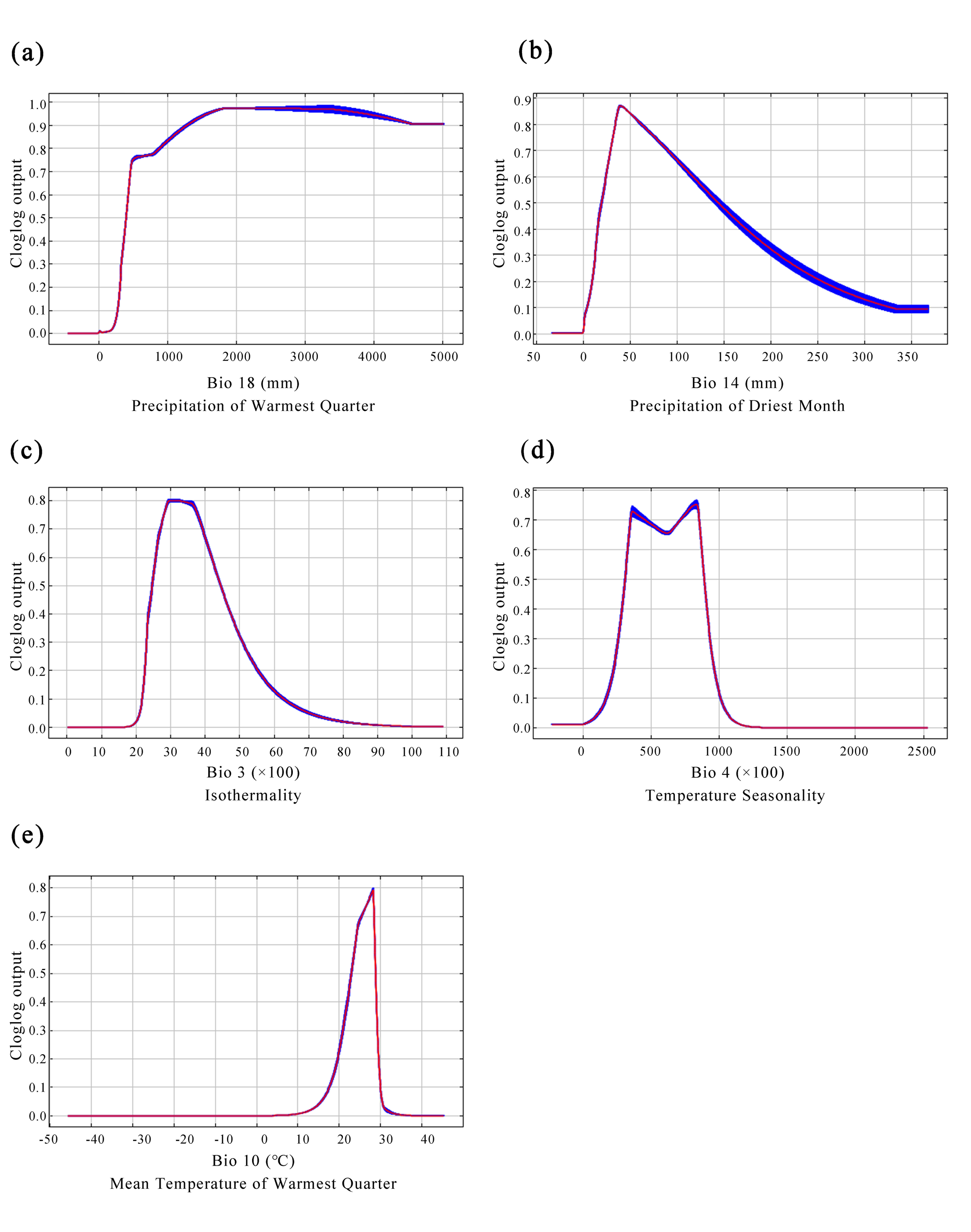

Supplement: Supplementary file 1 — Figure S1. Response curves between probability of presence and environmental variables. [file ECE3-14-e70435-s001.tif]
